# lab.js: A free, open, online study builder

**DOI:** 10.3758/s13428-019-01283-5

**Published:** 2021-07-28

**Authors:** Felix Henninger, Yury Shevchenko, Ulf K. Mertens, Pascal J. Kieslich, Benjamin E. Hilbig

**Affiliations:** 1 Mannheimer Zentrum für, Europäische Sozialforschung (MZES), University of Mannheim, A5, 6 (section A), 68159 Mannheim, Germany; 2 University of Koblenz-Landau, Landau, Germany; 3 University of Konstanz, Konstanz, Germany; 4 Heidelberg University, Heidelberg, Germany

**Keywords:** Experiment, Online data collection, Software, Open source, JavaScript, Open science

## Abstract

Web-based data collection is increasingly popular in both experimental and survey-based research because it is flexible, efficient, and location-independent. While dedicated software for laboratory-based experimentation and online surveys is commonplace, researchers looking to implement experiments in the browser have, heretofore, often had to manually construct their studies’ content and logic using code. We introduce lab.js, a free, open-source experiment builder that makes it easy to build studies for both online and in-laboratory data collection. Through its visual interface, stimuli can be designed and combined into a study without programming, though studies’ appearance and behavior can be fully customized using html, css, and JavaScript code if required. Presentation and response times are kept and measured with high accuracy and precision heretofore unmatched in browser-based studies. Experiments constructed with lab.js can be run directly on a local computer and published online with ease, with direct deployment to cloud hosting, export to web servers, and integration with popular data collection platforms. Studies can also be shared in an editable format, archived, re-used and adapted, enabling effortless, transparent replications, and thus facilitating open, cumulative science. The software is provided free of charge under an open-source license; further information, code, and extensive documentation are available from https://lab.js.org/.

## Introduction

The meteoric rise of the Internet over the past decades (The World Bank, 2016) has provided vast opportunities for behavioral science. Thanks to the access to larger, more diverse samples, it promises more flexible and economical research, and more robust findings as a result (e.g., Reips, 2007; Woods, Velasco, Levitan, Wan, & Spence, 2015). Because the browser is now a ubiquitous communication tool, data collection can take place in a multitude of settings, ranging from mobile devices in the field to the comfort of participants’ sofas, as well as the more controlled context of established laboratories. In addition, the ease with which data can be shared over the Web holds the potential to transform science by enabling the free exchange of materials and data and the transparent documentation of studies (Nelson et al., 2017; Nielsen, 2011).

The potential of browser-based and online data collection, despite these strong arguments in their favor, has yet to be realized to its full extent: The specialized knowledge required to construct and deploy online studies has limited their adoption—researchers had to program studies from scratch or rely on pre-made templates. Technical restrictions limited the accuracy of presentation and response times; proprietary tools hampered the exchange, re-use and extension of paradigms.

Our goal herein is to make available this untapped potential, and to increase the accessibility and usefulness of browser-based and online research by providing an open, general-purpose study builder—lab.js—in the spirit of widely used laboratory-based software (Mathôt et al., 2012; Peirce, 2007). It is designed to be easy to use without prior technical knowledge, but fully customizable and extensible by advanced users. It is built to integrate with the existing ecosystem of tools for online research, and to make use of the full capabilities of modern browsers for interactivity. It also provides excellent timing performance across systems, addressing a major concern in online experimental research (Hilbig, 2016; Semmelmann & Weigelt, 2017a; de Leeuw & Motz, 2015). Our software is available free of charge, enabling the open archival, sharing, and replication of studies. Indeed, it is designed to facilitate and encourage the exchange and re-use of tasks and paradigms. Thus, we have endeavored to meet the practical needs of researchers as well as the requirements of a modern, open, transparent, and reproducible scientific practice.

In doing so, we extend previous work that has identified and partially addressed these issues. In particular, libraries such as jsPsych (de Leeuw, 2014) and QRTEngine (Barnhoorn et al., 2015) have greatly facilitated manual coding of studies by providing programming frameworks that automate stimulus display and response collection for many common paradigms. The QRTEngine in particular pioneered new methods for more accurate experimental timing, and jsPsych was the first to provide a set of templates for common research scenarios, making in-browser experimentation much more accessible. All of these previous libraries, however, require programming skills. JATOS (Lange et al., 2015) and Tatool Web (von Bastian et al., 2013) offer easy-to-use interfaces that vastly simplify the assembly and deployment of task batteries, though the paradigms themselves are still constructed manually. PsyToolkit (Stoet, 2017) presents yet another perspective, using a custom text-based syntax to generate questionnaires and experiments.

Our software combines and augments these previous technical advances, providing an easy-to-use graphical interface that follows familiar conventions established by many laboratory-based, offline study builders. Most typical paradigms can be constructed using a what-you-see-is-what-you-get approach, without requiring programming or relying on a set of pre-made templates. At the same time, this ease of use does not place restrictions upon researchers looking to customize studies in full: The technical foundation of all studies is exposed, and can be accessed to adapt a study’s appearance and behavior: In principle, any content that can be included on a website can also be used within a study. Thus, while the graphical interface provides an accessible means of designing studies, customizability of the underlying logic vastly expands its capabilities and enables arbitrarily complex stimuli and experimental designs.

The software is the result of our own requirements and experience, and in constructing it, we have endeavored to meet the demands of modern experimental research: lab.js is freely available, and thus accessible regardless of resources. It is open as code, thereby amenable to inspection, customization, and extension. Because of its open nature, studies created with it can likewise be shared freely, re-used, and adapted, facilitating cumulative, open science. It is flexible, in that it accommodates the full range of behavioral research, from psychophysical studies to questionnaires. Finally, it can be applied across contexts, in a laboratory setting as well as directly on participants’ devices; there need only be a browser present.

## Building experiments in lab.js

*The following sections are adapted from the online reference and tutorial, which provide more in-depth information about the library as well as extensive introductory videos. The full documentation is available from the project homepage at* https://lab.js.org/.

Studies in lab.js are assembled from *components*, building blocks that, together, make up a study. These can be screens that present stimuli, sequences thereof, or more complex blocks that combine and repeat parts of a study. The lab.js *study builder* provides a visual interface for designing individual components and combining them into a larger experiment. The builder can be used in any recent browser without installation. It, too, is linked from the project homepage, or can be accessed directly by navigating to https://lab.js.org/builder.

For the purposes of this tutorial, we will focus on a variant of the classic *Stroop paradigm*, introduced by Stroop (1935). The most common version of the task presents color words in different hues, and instructs participants to identify the hue and disregard the semantic content of the word itself (see also MacLeod, 1991; Strauss, Sherman, & Spreen, 2006 for variants and more recent applications). The *Stroop effect* refers to the stable finding that this judgment takes longer when the two stimulus dimensions conflict, for example when the word red is shown in the color blue. This paradigm will serve as an example for the remainder of this tutorial, as its trial structure with crossed factors can readily be adapted to many other experimental tasks.

### Using the interface

**Fig. 1:**
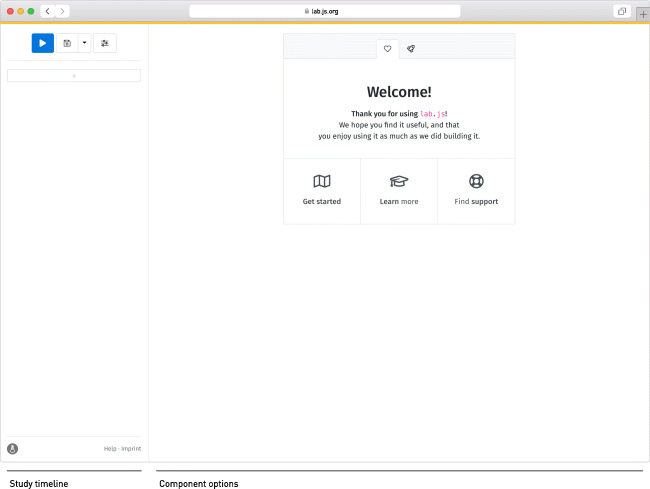
Initial view of the builder interface. After opening it for the first time, the study is empty

A first-time visitor to the interface will find an empty study waiting to be filled (Fig. 1). The welcome screen offers a variety of examples and templates to build upon, which we warmly invite users to explore; for the purpose of this tutorial, we demonstrate the construction of a study from scratch. Our first task, therefore, will be to add the initial content to the nascent study.

**Fig. 2:**
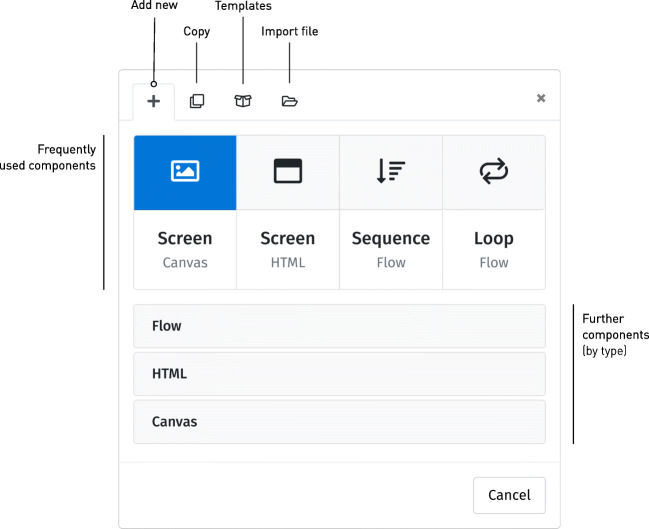
Adding a new component. The dialog box offers different types of component to be added, with four frequent choices directly accessible, and additional component types sorted by family in the drawers below. The tabs at the top provide additional capabilities: Duplicating an existing component, adding a new component based on a template, or importing one from a local study file (see ‘re-using components’ below)

The interface is divided into two panes: The pane to the left represents the study’s overall structure, providing access to the study’s constituent components, whereas the right represents the details and settings of any selected component. New components are added to the study’s structure using the plus button on the left-hand side of the interface. Clicking this button reveals a dialog offering a selection of various component types to add (Fig. 2). We revisit the different options below; for now, our first selection will be the leftmost: A *screen* of the *canvas* type.

### Designing screens

**Fig. 3:**
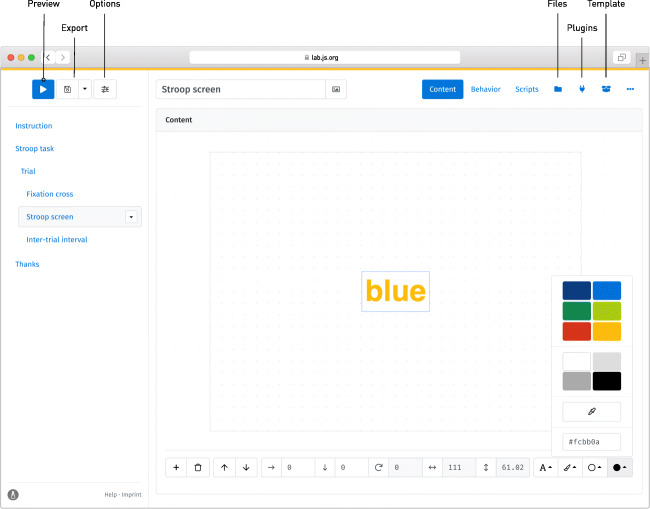
The visual editor in action, assigning a color to a piece of text. To the left of the interface, the Stroop screen has been selected for editing; its contents are visible on the *right*. Using the toolbar toward the top, the component’s name can be modified, and the editor below provides a preview of the later stimulus. The *blue border* indicates that the text has been selected; any changes made using the toolbar below are applied to it

Having added the new screen component, its settings become visible on the right-hand side of the builder (Fig. 3). Canvas-based screens are designed using a visual editor that provides a direct preview of the later display. Through the toolbar below the editor, text, images, and other visual content can be added using the button with the plus symbol and then moved and re-sized directly within the preview. The toolbar also provides access to an element’s precise location, color and (if applicable) further options. For the purpose of our Stroop task, we might add a single word in the center of the screen, select a color for it, and increase the font size and weight. With this basic screen complete, a click on the blue *preview button* in the top left toolbar shows the task as a participant will experience it, and provides a first glimpse of the study in progress.

Working towards a single trial, our next step is to extend the study by adding further screens of the same type: A screen with a centered plus sign prior to the Stroop screen serves as a fixation cross, and an empty inter-stimulus interval follows it. As before, new components are added to the study structure on the left-hand side, where their vertical order represents the temporal succession during the study. Briefly hovering the cursor in the intended location of the new component, in this case above and below the existing screen, once more reveals the button for inserting components. Previewing the study at this point, however, shows that the task never progresses beyond the initial fixation cross; clearly another change is in order.

### Changing components’ behavior

**Fig. 4:**
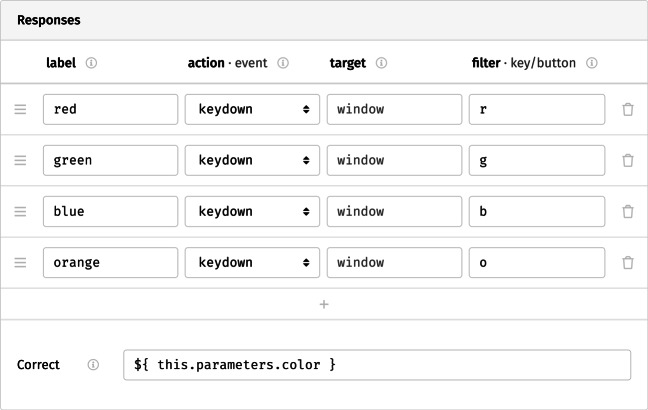
The possible responses available on the Stroop screen, with each color represented using a different key. Below, the correct response has been defined using a placeholder (see text for a detailed description)

Beyond their contents, further options for any selected component can be set through the different tabs listed at the top right of the interface. The *behavior* tab determines its behavior during the study: Here, the topmost set of options provides a visual representation of the component *timeline* on which additional events and stimuli can be scheduled (e.g., sounds), as well as a timeout after which the study moves on to the next component. For the fixation cross and the inter-trial interval, we would like screens to disappear after a fixed amount of time (we might choose, for example, a 500-ms duration for the fixation cross and 1000 ms for the inter-stimulus interval). For the Stroop screen itself, the participant’s response triggers the transition; therefore, the set of permissible responses must be defined (Fig. 4). In the *responses* grid, the first column, label, represents the qualitative meaning applied to any response. In our case, the available options are the different colors in which the word might be shown. The next columns request that we select between different actions that a participant might take to make their response. In our study, participants indicate their answer by pressing one of several keys. Thus, the event that corresponds to a response is the keydown. This is not the only option: mouse-based response modes are also available, and responses can be triggered specifically when a button or key is pressed or released. The next column, target, would allow us to limit responses to specific parts of the screen, for example by restricting mouse clicks to predefined content or screen areas. By leaving the field empty, we allow responses anywhere onscreen. The final column allows us to filter events further, in this case by key: Entering a single letter assigns the corresponding key to the response in question (multiple alternative buttons can also be mapped onto the same response, by separating the keys with commas). With timeouts and responses in place, the study now runs through a single trial when previewed.

### Flow control

A single trial is, of course, only the first step toward a useful study: A complete Stroop experiment will contain many trials, each repeating the screens we have just built while varying word and hue. We could duplicate our screens and change their content manually to create a longer study, however this would be an effortful and tiresome endeavor, and limit the study to a static order of stimuli. To more easily create an experimental task, *flow control* components allow us to go beyond the linear sequence of screens we have encountered so far. We will use this type of component, first, to group the existing screens into one unit. The software can then repeat this grouped set of screens to create a recurring trial structure automatically. Our final task will then be to add variation between these repetitions.

The most basic flow control component is the *sequence*, which combines a set of successive components. A sequence is added to the study structure in the same way the screens were (cf. Fig. 2), and other components can be nested within by drag and drop. When this is complete, a slight indentation indicates that multiple screens have been combined into a larger unit, in our case into a single trial (as in Fig. 3). Though it is required for the next step, at this point, the grouping is for our convenience only, and invisible to participants.

**Fig. 5:**
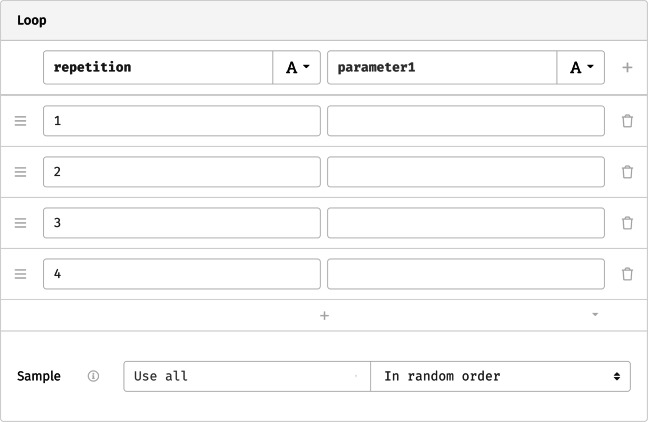
Minimal settings for a loop that only counts repetitions

A *loop* repeats a single component (a screen, sequence, or another loop) multiple times. Like the sequence, it accepts nested content. Its main options concern the number of repetitions and the attributes that define each one. These are represented as a grid (Fig. 5), with every row representing a repetition of the loop’s contents, and every column a parameter that varies across iterations. In the most basic case, we might name one column repetition and assign a number to every cycle. Previewing the study at this point shows that the trial is repeated the requested number of times—but it is repeated verbatim, without variation in either color name or hue, thus lacking the manipulation at the core of the Stroop paradigm.

### Defining parameters

**Fig. 6:**
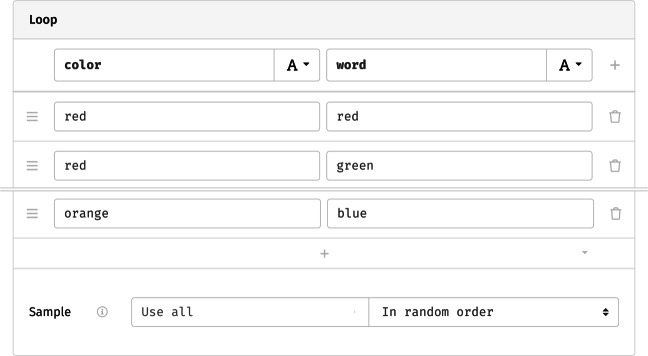
Loop parameters for the Stroop task (abridged). In the columns, the presented word and its hue are defined as two string variables; each *row* represents the set of values that determine one iteration. *Rows* are shuffled before presentation, resulting in a random ordering during the study

To add meaningful variation across loop iterations, we can define *parameters* that change between repetitions, and set their respective levels. For the Stroop task, these are the presented word and its color. To implement this design, we might name two columns in the loop’s grid word and color and fill them with the desired combinations (Fig. 6). The plus button at the bottom of the grid can be used to add further rows as needed, generate a larger grid from a given design, or to load and save the entries to a csv file. Lastly, as the option below the grid indicates, the software will run through all repetitions in random order, though this could be changed by limiting the number of trials or switching to one of the other available randomization schemes.

Even with this addition, the screen remains constant: We’ll need to include the varying parameters in the screen content so that the display and behavior reflect the experimental manipulation.

### Using placeholders

**Fig. 7:**
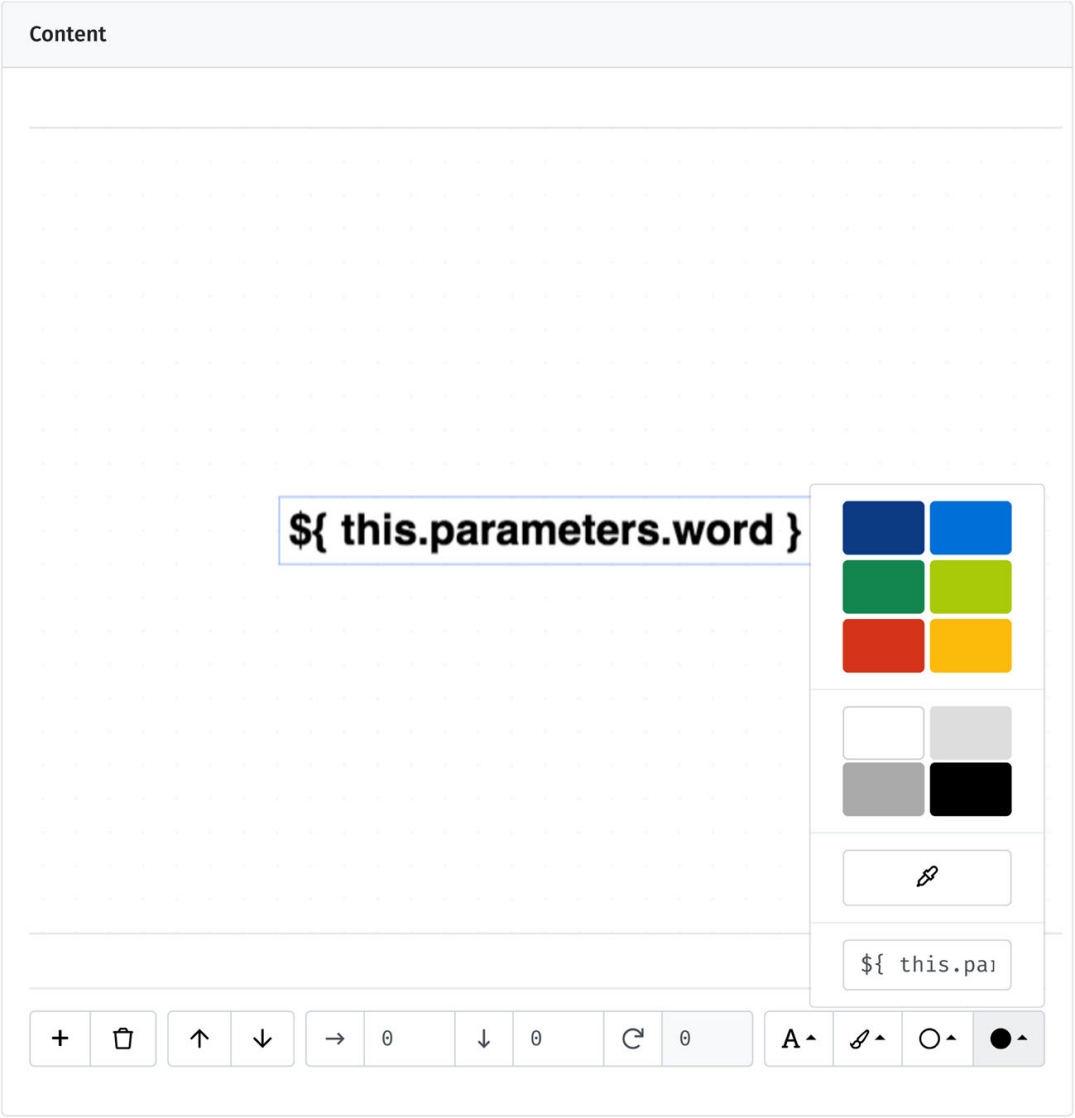
Final version of the Stroop screen, including placeholders in place of the text content and hue (at the bottom of the color selector)

To mark insertion points for varying content, lab.js uses *placeholders*, temporary stand-ins that are replaced by other data during the study. Placeholders are defined using a dollar sign and curly braces, ${}, where an expression between the brackets represents the data that is substituted as the component is prepared. To make use of the previously defined parameters, for example, we can insert ${ this.parameters.word } in place of the fixed screen content, as a placeholder for the currently static word shown during every trial (Fig. 7). Similarly, by replacing the color code in the toolbar’s fill option with ${ this.parameters.color }, we can vary the word’s color. As a result, color and content now change dynamically across trials. At this point, we have constructed a complete within-subjects experimental paradigm, entirely without code. Many like it can be assembled with similar ease.

Placeholders are central to studies in lab.js, and can be used in most options throughout the builder interface. For example, because the goal of the task is to name the color, we might insert ${ this.parameters.color } in the *correct response* field on the behavior tab (cf. Fig. 4). Here too, the corresponding value is substituted, and the accuracy of the response coded accordingly.

### Previewing data

**Fig. 8:**
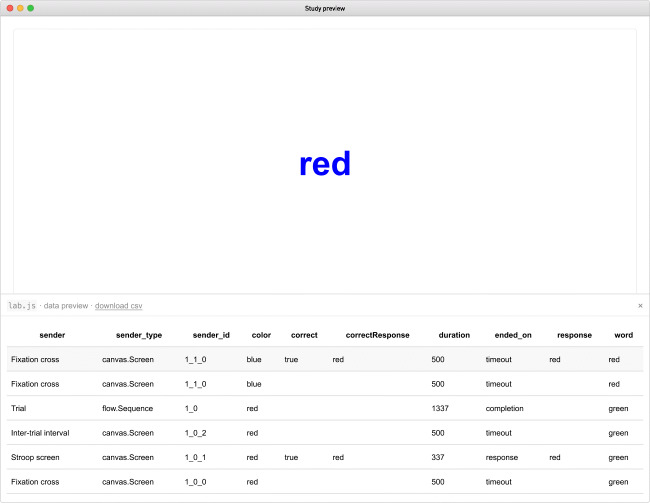
The study preview mode, showing a trial running in the background, with the data overlay visible at the bottom of the screen. In the table, the study’s progress is reflected in reverse order: The previous screen was the fixation cross; before it, the participant completed a trial sequence. The columns contain metadata, timestamps, and parameters as well as the collected response. The topmost row (*shaded*) represents the study’s current ‘state‘, and repeats the last entry in every column: Even though the fixation cross did not elicit a response and terminated after a timeout, the last trial’s decision is still available, and will remain so until replaced by the upcoming response

Data are collected automatically as the study progresses. In the study preview, the accumulated data can be accessed through the small button in the lower right-hand screen corner. The button reveals a data grid (Fig. 8) where different variables are collected in columns; rows represent a single component each. Every screen, sequence or loop adds a line the instant a participant moves beyond it. In the preview (but not the final dataset), the rows are reversed, so that the latest entries are added on top, where they can be compared directly to the current screen content.

The entries in the sender column reflect the origin of the data through the name of the corresponding component; the column sender_type indicates its type. Further columns include timing information, the response given by the participant, and the parameters set by the design. If a correct response has been defined, it is included, as is an indication of whether the recorded response matches this standard (as a Boolean value, true or false).

### Experiment state and feedback

The topmost, shaded row in the data preview represents the latest entry in each column, or the study’s current *state*. Through the state, data from previous components is accessible until overwritten by a new entry in the same column. For example, in our task, the last observed response persists until the next stimulus is shown, because the intervening components do not accept or store responses. This is often useful, particularly when providing feedback regarding previous answers.^1^

The study’s *state* can be used within placeholders, in a manner analogous to the parameters introduced above. For example, we might include ${ this.state.correct } on the inter-trial screen to show the accuracy of the last response and provide feedback to participants. However, if we were to try this, the screen would remain empty. This is because lab.js, in order to maximize performance, attempts to prepare and render all screen content as early as possible, ideally as the page is loading.^2^ Thus, by default, screen content is fixed entirely before participants start interacting with the study, and data generated later is not available for inclusion in components. To remedy this, individual components can be set to *tardy* mode by checking the corresponding box on the *behavior* tab. Activating this option on a component means that it is prepared only just prior to its presentation, allowing it to reflect the latest collected data, though at the expense of some further processing (and potentially a minuscule delay) during the study. Activating tardy mode on the inter-trial screen makes the feedback visible—the screen now indicates the veracity of the response through the values true and false. Admittedly, this Boolean value is not the most friendly feedback, but thankfully, it is also not difficult to replace.

### Logic in placeholders

Placeholders can contain any JavaScript expression, so that it is possible to include small programs directly in the screen content, or in any other option that supports placeholders. So far, we have retrieved values from variables and included them verbatim, but expressions give us the opportunity to perform further computations based on state and parameters. For our example, we might want to translate the binary values into more helpful feedback by replacing the Boolean values with friendlier messages. A *ternary expression* helps us achieve this, by switching between two outcomes based on a binary variable. It consists of three parts, a binary criterion, and two values that are substituted depending on whether the condition is met or not. For example, ${ this.state.correct ? 'Well done!' : 'Please try again.' } evaluates into the message ‘Well done!’ after correct responses, whereas the message ‘Please try again.’ is shown following incorrect answers.

This use of expressions is not limited to substituting content; it can be used within any placeholder. For example, we might provide graphical feedback by switching the color of a circle between green and red depending on the participant’s response. Nor is accuracy the only possible switching criterion: We could similarly instruct participants to respond faster by comparing the value of this.state.duration to a fixed threshold. Thus, combined with expressions, placeholders provide a very general and flexible mechanism for varying all of a study’s content and behavior.

### Using html

Although the visual interface is undoubtedly convenient, the browser offers more options for defining content. Indeed, most information on the Web is not defined as a fixed visual layout, but using the Hypertext Markup Language, html. This technology allows studies built with lab.js to draw upon the manifold options and resources for content and interaction design available to any web page, which extend far beyond the capabilities of many classical experimental tools. lab.js supports html-based screens through a dedicated component type (second from the left in Fig. 2), and studies can combine both as required.

Defining screens using html does not fix their layout precisely as the visual editor does; instead, screen content is represented as text, and augmented by *tags* that reflect the semantic purpose of any part of the page. To provide a more concrete example,  indicates that the main content of a screen is the word blue. The tags, enclosed in angle brackets, are not shown to the user. Instead, the browser takes into account the additional semantic information they provide, and chooses an appropriate visual representation. Content defined using html may consequently vary in its exact layout across different devices and browsers, adapting to screen sizes, zoom settings, resolutions, and aspect ratios. In comparison to the visual editor, the code-based nature of html takes a small amount of time to learn, which its flexibility quickly repays. To support users, the built-in editor completes tags as they are entered, highlights potential mistakes and suggests remedies.

**Fig. 9:**
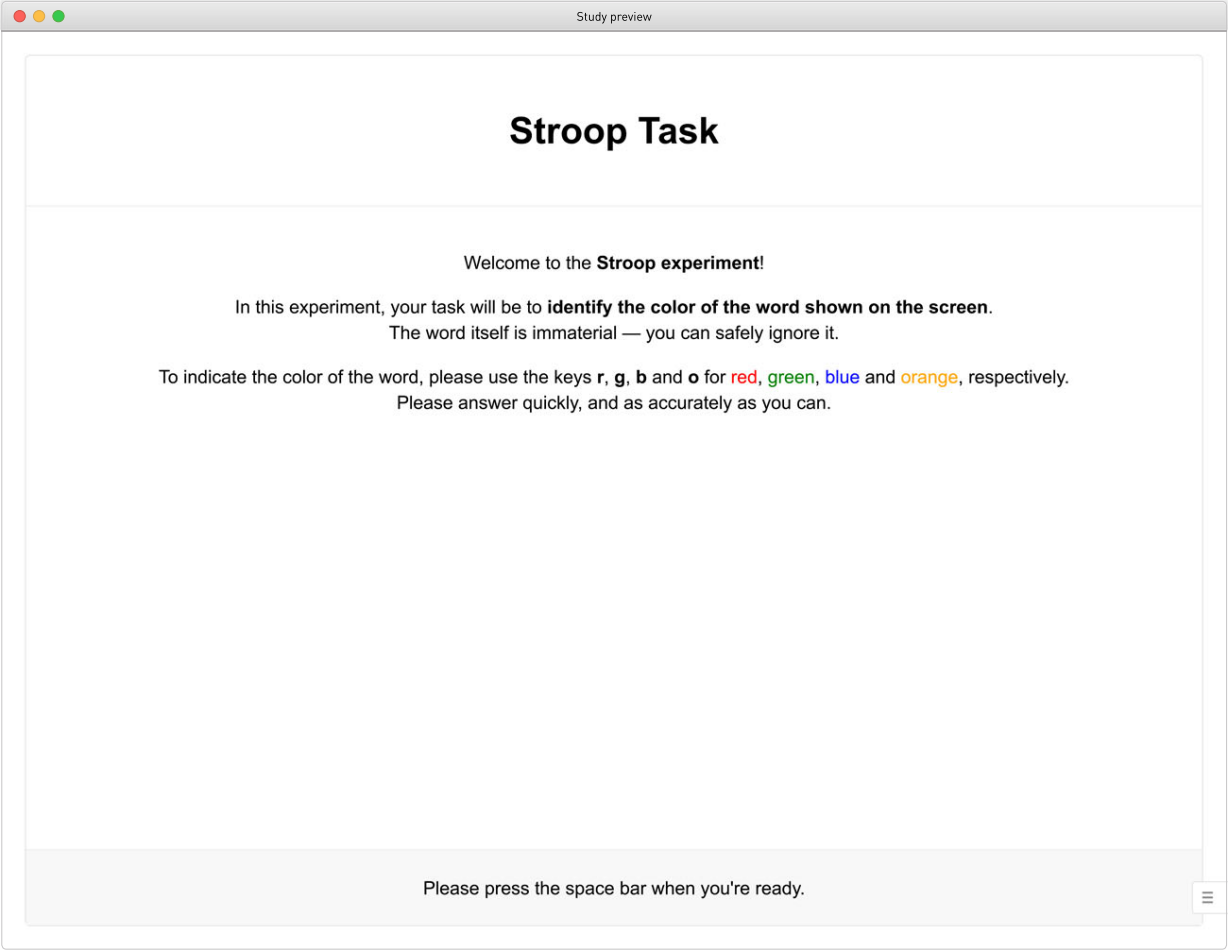
Three-tiered html-based instruction screen using ,  and  elements

Screen design presents another common hurdle for beginning online experimenters, since it requires formatting instructions defined in the css language. In this regard, too, lab.js assists researchers by providing built-in defaults for commonly used layouts. For example, a three-tiered vertical structure can be designed quickly by adding ,  and  tags to a screen and placing the respective content within them; the built-in styles will automatically provide an appropriate screen layout (e.g., Fig. 9). To facilitate placement and alignment of content, the library also provides a range of convenience css classes.^3^ As an example, the content-vertical-center class centers content on the vertical axis (alternatively, content can be moved to the top and bottom vertically, and horizontally to the left, right and center).

Dynamically generated content can also be embedded into the html syntax using placeholders. Thus, a minimal html equivalent to the graphically constructed screen we constructed above might be the following: 
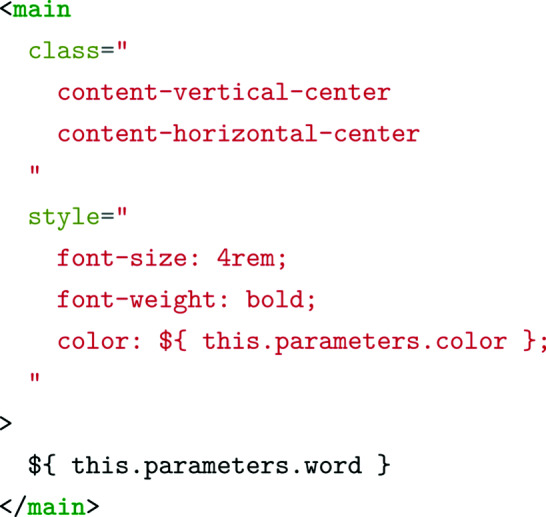


The free combination of html- and canvas-based screens allows researchers to mix different content types at their convenience. For example, we have found it useful to define instructions using html so that their content can adapt to the screen size, while designing stimuli using the canvas for maximum performance and consistent presentation across devices.

### html forms

An additional advantage of html is its ability to represent forms and questionnaires, making lab.js useful beyond purely experimental research. This is supported through form components, which capture and process data collected in forms.^4^ Their content is also defined using html,^5^ so that a minimal questionnaire might be represented by the following snippet: 
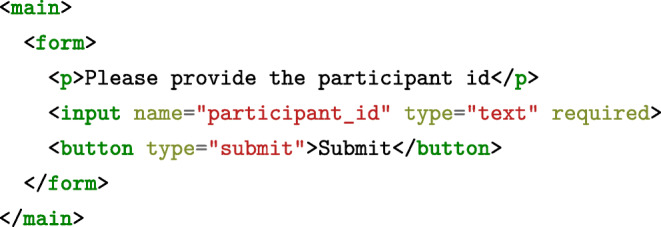


The above code translates to a basic form onscreen, containing a single text-based input field and a button to submit the data. The form component extracts all data automatically upon submission: In this case, the entry is saved in the participant_id column; any additional fields would likewise be included in the participants’ datasets.

To ensure consistency of the collected information, validation logic can be applied. In our example, the required attribute dictates that the field contain a value, and a participant will only be able to move further after filling it. Beyond the mere presence of a value, more complex validation is possible within the html code, for example by constraining entries to a given input type (e.g., number, email, date, etc.) or comparing them to a predefined pattern.

## Study deployment, archival, and re-use

With the construction of the study, of course, the scientific work has only just begun. Data collection and the archival of a study’s materials are further, central steps in the scientific process that lab.js makes easier and more efficient.

### Saving a study to disk

As with most other experimental software, studies constructed in lab.js can be saved to a single file that contains all content, settings, stimuli and auxiliary files. This file can be downloaded using the corresponding button in the toolbar (Fig. 1), and is best suited for development and public archival. Using the dropdown menu next to the save button, an experiment file can be re-opened later for inspection and further modification.

### Deploying a study for data collection

For a study to be truly useful, it must run beyond the confines of the builder and the experimenter’s device, and be made accessible within a laboratory or publicly on the Internet. This, too, previously demanded specialized technical knowledge, and therefore presented a challenge for researchers considering online data collection. Depending on the project goals and the available infrastructure, lab.js offers a wide and growing range of options for data collection, all designed to vastly simplify the previously complex task of hosting studies. All deployment options are likewise available from the dropdown menu next to the save button.

The most basic option is *offline data collection*, which bundles all necessary files to run the study entirely without external infrastructure. This export option results in a zip archive that contains the study as visible in the builder preview, pre-configured to save all collected data as a csv file at the end of the study. Besides facilitating local testing and data collection, this type of bundle can easily be shared in a preregistration or archived alongside data and other materials, so that the exact appearance and behavior of a study are preserved and documented.

Data collection over the Internet is the common feature of all further export options. Researchers looking for an effortless hosting option might opt for a *cloud deployment*, placing hosting and data storage in the hands of a highly reliable external service. Studies can be transferred to an external provider^6^ directly from the builder in a matter of seconds, and data collection can start immediately.

Where full control over the data collection process is required, the php *backend* bundle can be used to install a survey on most, if not all, common web servers. This option produces a zip archive which, extracted on a php-enabled webspace, fully automates data collection: Data are continuously sent from the client and gathered in a database as participants complete the study.^7^

Studies created with lab.js also integrate with external tools as part of a larger data collection project. Another export option creates the code required for integration in *survey tools* such as the proprietary services Qualtrics (Qualtrics, 2016) or SoSci Survey (Leiner, 2014), and open-source alternatives like the powerful survey frameworks Formr (Arslan et al., 2020) or LimeSurvey (Limesurvey GmbH, 2018). Beyond that, the builder provides a direct export to The Experiment Factory (Sochat et al., 2016; Sochat, 2018), which is an open-source framework for assembling and hosting batteries of tasks in fully reproducible containers, as well as jatos (Lange et al., 2015), Open Lab (Shevchenko & Henninger, 2019), and Pavlovia (https://pavlovia.org/). These easy-to-use, comprehensive, open-source study hosting platforms not only make study hosting, recruitment and data collection easy, but provide further features such as (in the case of jatos) real-time interaction between participants and coordination with crowdsourcing services such as Amazon’s Mechanical Turk.

Through all of these deployment options, we aim to support a wide range of data collection scenarios, so that lab.js can be used by researchers regardless of their technical experience and the infrastructure at their disposal. Across all alternatives, we automatically implement *best practices for online research* wherever possible, with the least amount of effort on part of the user. For example, cloud and server deployment options are configured to support the multiple site entry technique, through which participant populations can be distinguished by the URL through which they access the study (Reips, 2002). Likewise, the software automatically captures information provided by external recruitment services, such as worker and task id s generated by Amazon Mechanical Turk (cf. Stewart, Chandler, & Paolacci, 2017). Where external files are used, their paths are obfuscated so as not to reveal the experiment’s structure.

### Re-using components directly

Beyond the publication and re-use of entire studies, lab.js is built to facilitate the recombination, extension, and exchange of individual components or larger parts of an experiment. Screens, forms, or entire tasks are designed to be self-contained and easily transferable between studies.

Every part of an experiment can be independently exported as a stand-alone file and re-imported into a new study, preserving all content and settings. This option is available from the drop-down menu next to any selected component in the study overview. The resulting file can be imported into a study from the dialog box for adding new components, or used as starting point for an entirely new project.

### Creating task templates

To facilitate the re-use of more complex paradigms, parts of a study can also be designated *templates*. This reduces a set of nested components into one unit, creating a single component that encapsulates an entire task, and can be easily dropped into other studies. For example, the Stroop task we constructed earlier could, once completed, be condensed into a single template component, making it much easier to reuse it, for example as part of a larger test battery.

If a component is marked as a template, all of its contents and options are hidden and thereby protected from inadvertent modification. In their place, a much simpler interface provides access to only the settings relevant to the task. For example, template authors can allow users to adapt the number of trials, response buttons, instruction texts, and other parameters without requiring them to delve into the details of the task. Any values set by users are available inside a template through the parameter mechanism outlined above, and can be inserted inside placeholders.

Through the template mechanism, lab.js bridges the gap between manual programming, which offers control over every aspect of a study, and entirely template-focused tools that limit researchers to a set of predefined tasks or stimuli. Using templates, more advanced users can package technically complex paradigms into easy-to-use units that can be reapplied without the expertise and effort that were necessary to create them. This, however, does not hinder customization and adaptation—by exposing the relevant settings, a task can be adjusted to match the needs of a research project without requiring detailed knowledge of its innermost workings. Because the template setting is reversible, the accessibility of a task bundled as a template does not preclude in-depth inspection and modification: Paradigms can be handled and modified at multiple levels of abstraction or technical detail, suiting the needs of the individual researcher.

## Timing performance

A common concern of researchers considering online data collection has been the accuracy and precision of presentation and response timing, especially for fast-paced experimental paradigms. Empirical validation studies have found that browser-based stimulus display and response collection incurred lags and variability both within a given browser and across different combinations of browser and operating system (e.g., Reimers & Stewart, 2014). Though many phenomena are demonstrably robust to any measurement inaccuracy introduced both by moving from dedicated experimental software to browser-based data collection and gathering data outside of the controlled laboratory setting (Semmelmann & Weigelt, 2017a; de Leeuw & Motz, 2015; Hilbig, 2016; Crump et al., 2013; Simcox & Fiez, 2014), considerable room for improvement has remained with regard to both accuracy (denoting freedom from bias or lag) and precision (freedom from measurement noise, Plant & Turner, 2009). With lab.js, we build and improve upon previous approaches to browser-based experimentation, reducing both lags and measurement noise, and further approaching the performance of native experimental software (see also Henninger, Schuckart, & Arslan, 2019).

The first prerequisite for precise time measurement is exact timekeeping. Our framework consistently uses *high-resolution timers* that provide sub-millisecond precision for all measurements, following Barnhoorn et al., (2015). This is a simple, but effective improvement over previous in-browser timing methods that truncate timestamps at the millisecond level by default.^8^

A second imperative for precise timing is that measurements are synchronized to the display refresh. Failing to do so results in added noise because time measurement might start before or even after a stimulus has been presented. Therefore, *frame synchronization* is commonplace in dedicated, native experimental software (cf. Mathôt et al., 2012). In lab.js, all timer onsets are aligned to the browser’s animation frame cycle, which closely tracks the underlying graphics hardware (Barnhoorn et al., 2015). Presentation times are likewise synchronized to browser’s screen update rate: An adaptive algorithm monitors the current rendering performance and presents new content with the frame that most closely matches the intended display duration. This provides a considerable improvement over the typically used setTimeout function, which is prone to overshooting any specified duration and thereby adding lag.

The final element to high-performance timing is an *optimized rendering engine* that minimizes delays in stimulus presentation. Here again, lab.js improves upon previously available tools, adopting strategies formerly found only in native experimental software: It reduces computation during the study as much as possible, pre-loading and preparing stimuli prior to their presentation (a prepare-run-strategy, cf. Mathôt et al., 2012). For screens constructed using the visual editor, the canvas-based rendering engine provides flexible, high-performance graphics capabilities by removing the computationally expensive layout calculations required for HTML content. Users can further minimize the amount of content that the browser needs to re-render during the study through Frame components, which provide a constant frame of html content around a changing stimulus, thereby avoiding large changes to the document and the corresponding costly layout recalculations. For example, in our Stroop task, we might extract those parts of the screen that remain constant during the task into a frame and place the stimulus loop inside, varying only the main content between screens while leaving its surroundings (e.g., instructions) in place. Thereby, only the actual stimulus is exchanged between screens instead of re-drawing the entire display with every change, however minor. Using the canvas.Frame component to enclose a set of canvas-based screens provides a common  element across all screens. This eliminates changes to the html document entirely, further increasing rendering performance.

**Fig. 10:**
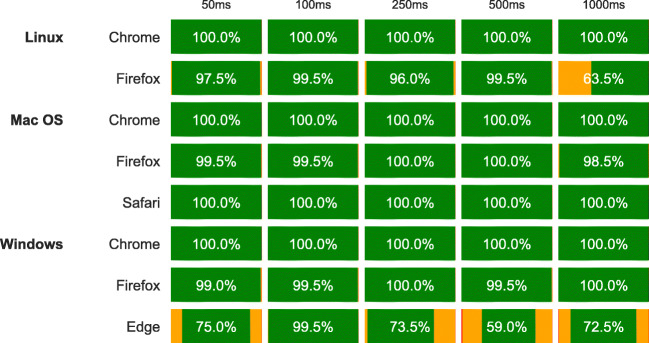
Timing validation results for stimulus presentation, in percent of target frames hit across simulated durations, browsers, and systems. The *green areas* represent the proportion of exact matches, *orange areas* are one frame to early or to late, and *red areas* two frames or more (only the case for Internet Explorer Edge, in less than 1% of the two longest presentation intervals). See also https://lab.js.org/performance/ for the most recent timing results

**Fig. 11:**
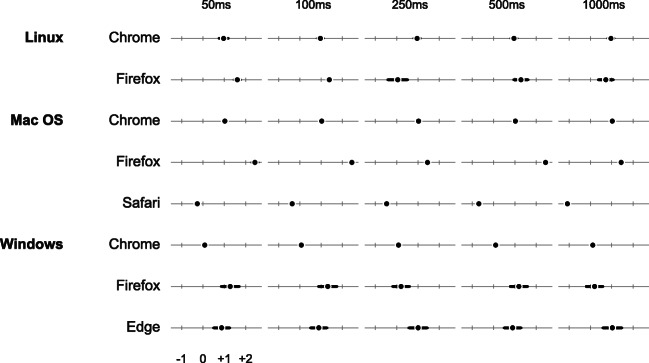
Timing validation results for response time measurement across browsers and systems. *Dots* represent the mean difference between simulated and captured response times in frames, and *bars* the observed standard deviation. See also https://lab.js.org/performance/ for the most recent timing results

With all of these features in place, the practical performance of the library remains to be demonstrated empirically. To this end, we conducted a technical validation study similar to that reported by Mathôt et al., (2012) and Reimers and Stewart (2014). In particular, to capture the software’s performance in isolation, we measured the actual duration of stimuli output by the computer, and used external hardware to simulate precisely timed responses, which we then compared to the response interval measured by our software. In our tests, we created a continuous series of rapidly changing screen content, creating a load that likely exceeds the demands of many typical experiments (Garaizar & Vadillo, 2014). This we repeated across a range of common browsers and operating systems to evaluate the consistency of results (further details regarding our method are provided in the Appendix).

Figures 10 and 11 summarize our validation results. In a nutshell, presentation intervals were consistently met across browsers and operating systems. Chrome and Safari always matched the intended stimulus duration exactly. Firefox met this criterion in more than 98% of our measurements on Windows and mac os, with somewhat reduced performance on Linux. However, Firefox never deviated more than a single frame from the target interval. Internet Explorer Edge showed a considerable increase in single-frame deviations, and 0.3% of measurements two or more frames off the preset duration. However, the excellent performance across all other browsers demonstrates that this is specific to IE, and one might expect this browser to catch up with its competitors as it matures.^9^ The overall result thus demonstrates that lab.js offers extremely precise stimulus timing capabilities, with the best-performing browsers approaching the level of popular native experimental software (Mathôt et al., 2012; Garaizar et al., 2014; Garaizar & Vadillo, 2014).

Regarding response times, our results show somewhat greater variability across browsers. Most consistently overestimate response latencies by between one and two frames (16.7 to 33.4 ms), with fairly little noise (the maximum sd we observed was 7.4 ms, in Internet Explorer Edge at 1000-ms response latency). Chrome stands out not only for its small measurement variability across operating systems, but also for its consistent lag of almost exactly a frame on Linux and mac os, and around 1.5 ms on Windows. We fully anticipate that this result will improve further with browsers’ future development, and provide more detailed and up-to-date information at https://lab.js.org/performance/.

All this being said, we would like to emphasize that the standard of *absolute* timing accuracy and precision applied above, while well worth pursuing, is a very high one. In practice, the measurement noise for response times we report above is negligible in many common paradigms: Even for between-subject experimental comparisons, Reimers and Stewart (2014) show through simulations that a small increase in the number of participants makes up for any loss of power due to between-browser variations and a timing noise larger than the one we observed (see also Ulrich & Giray, 1989; Damian, 2010; Brand & Bradley, 2012). Similarly, within-subjects designs that focus on differences in response times between conditions (which we intuit are already common in paradigms that rely on response times) are insensitive to any consistent lag introduced in timing (see also Reimers & Stewart 2014, for an in-depth discussion). Our sole caution is that correlations between individual differences and absolute response times might be mediated by participants’ choice of browser (Buchanan & Reips, 2001), but again, compared to common variation in response times, we observe only very small differences between browsers. Should such concerns, however, become pressing, studies built in lab.js also translate naturally to a laboratory setting, which provides the opportunity to run the study in a browser with the desired timing characteristics, and on consistent hardware.

In sum, reviewing this pattern of results and particularly the timing performance that lab.js offers in combination with the most powerful browsers, we cautiously predict that further improvements are unlikely to stem from browser-based experimental software itself, but will result from browser and operating system advancements. Finally, we would like to note that all of these measurements are exclusively concerned with, and therefore purposefully isolate, our software’s performance: Beyond any timing inaccuracy introduced by the software, common peripheral hardware such as off-the-shelf keyboards and displays is likely to introduce further lags and measurement noise (e.g., Garaizar et al., 2014; Plant & Turner, 2009; Lincoln & Lane, 1980). These, however, apply not only to online data collection but also most laboratory settings, unless specialized response hardware is used.^10^ Though the variability of peripherals outside of the laboratory is likely to introduce a slight amount of additional variance, this is unlikely to affect qualitative findings except for the smallest of effects (Hilbig, 2016; Semmelmann & Weigelt, 2017a; Brand & Bradley, 2012).

## Technical underpinnings

The workhorse of stimulus display and data collection in lab.js is a custom JavaScript framework that governs all interaction with participants. In the workflow demonstrated above, the builder interface generates a JavaScript representation of the study, which is read and executed by the framework within participants’ browsers. The entire study logic thus runs on the client side, reacting immediately to participant input and eliminating network latency which would be introduced by loading new page content from the server between screens.

All components in a study can be customized through JavaScript code to fit the requirements of the scientific endeavor at hand. In the builder’s scripts tab, custom instructions can be attached to every component, to be run at specific points during its lifecycle. For example, when it is prepared, a component’s options might be adjusted depending on participants’ previous performance, enabling adaptive experimentation. When a component is run, the presentation can be extended beyond the default behavior, enabling, for example, interaction patterns within a single screen that go far beyond the standard stimulus-response pattern. In addition, code can be executed when the presentation comes to an end, for example to compute indices based on the collected responses. Similarly, because the library exposes the stimulus canvas and the html document directly via standardized browser interfaces, any content or library that can be included on a regular web page can also be added to an experiment built with lab.js. This ability to add custom logic during the study, and to draw from the rich ecosystem of the web, greatly increases the flexibility of our tool, allowing it to cater to applications yet to be envisioned, and to complete tasks we have not foreseen or implemented directly in the interface.

### Programming studies in pure JavaScript

Researchers interested in defining studies directly in code can use the underlying library independently of the builder for maximum flexibility. It exposes the same building blocks through a declarative, object-oriented interface which mirrors the graphical builder. To give a brief example, a basic canvas-based Stroop screen would be defined as follows: 
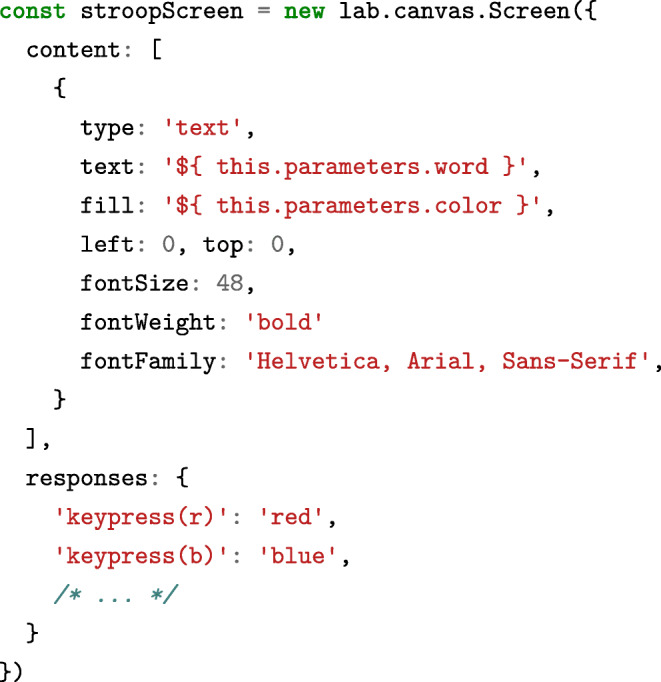


As in the builder interface, these basic stimuli can also be combined into superordinate components: 
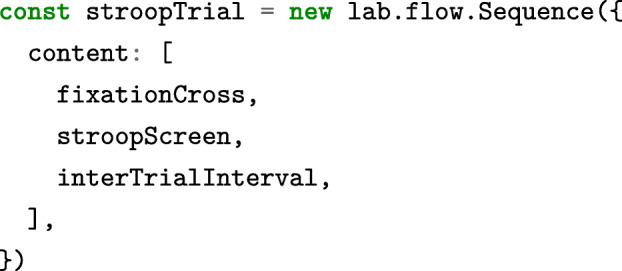


All of these components provide a consistent JavaScript interface. For example, a developer might write stroop Screen.run() to trigger the preparation and display of the screen defined above, which would show the stimulus and wait for one of the predefined responses. The exact same method can be applied to the stroopTrial to present a slightly more involved sequence of events. To include custom logic, instructions can be added to any component for execution at a later point in time through the command .^11^

On a technical level, all different components are linked through JavaScript’s inheritance mechanism, and adopt the vast majority of their behavior from the general-purpose lab.core.Component, extending it only as required by their specific use. For example, the lab.html.Screen component only inserts its html content into the page when it is run; most other logic, including the substitution of placeholders, is provided by the library core. In a similar way, and with very little effort, new functionality can be added to the library itself by creating custom components that perform specific tasks. In addition, a *plugin* mechanism exists to attach logic to any component in a study, regardless of its type. This is, for example, used to provide the data preview for all parts of the study.

We have taken great care to follow best practices for scientific software (Wilson et al., 2014) while developing lab.js: The code is available under an open-source license which allows for free extension and modification. All changes are tracked in the project repository, and a set of automated tests run across multiple browsers are applied to every one, ensuring continued stability and compatibility. We are confident that this infrastructure will greatly facilitate sustained development. Experiment files are automatically upgraded to the latest library version, incorporating changes and updates, while exported studies include all necessary files to support continued use and long-term archival.

## Discussion

lab.js provides an easy-to-use, visual interface for building browser-based studies, enabling efficient data collection both in the laboratory and online. The graphical builder makes the design of studies easy, while html, css, and JavaScript integration give researchers full control over their studies’ presentation and behavior. Its present focus is on experimental paradigms, for which it provides powerful, high-performance stimulus and response timing methods, but our library supports the full gamut of behavioral research, and can make full use of the powerful capabilities of modern browsers.

We believe that the advantages of a platform like lab.js are not limited to more efficient data collection: Because the web is an almost ubiquitous medium, and lab.js freely available, studies can easily be shared with colleagues before they are used in the field, facilitating collaboration within a project. Following publication, studies can be publicly archived, viewed, modified and adapted by interested researchers, who can build upon previous efforts and customize or extend existing studies without having to re-implement a paradigm in its entirety. Our software makes it easy to export parts of studies in an editable format for sharing and re-use, facilitating collaboration and cumulative science (Nielsen, 2011; Ince et al., 2012); completed studies can be similarly shared in archivable form, so that paradigms can be viewed and potentially replicated directly without additional software.

Of course, some lines of research may require dedicated native experimental software, particularly if they involve specialized external hardware. That being said, browser-based experimental software supplements and may in many cases supplant native experimental software, and its capabilities are continuously expanding (for example, physiological measurements and basic eye-tracking are already possible, see Semmelmann & Weigelt, 2017b).

Through lab.js, we have aimed to make available the power and flexibility offered by dedicated laboratory-based data collection software in the browser and on the web. We hope that it will enable a more efficient (and, keeping with the spirit of Mathôt et al., 2012, perhaps even fun) realization of behavioral science, and we would be proud to support the ingenuity and creativity of our fellow researchers. The internet has been the medium with the fastest growth in the history of humanity, and it continues to evolve rapidly. We hope to track these developments and incorporate future best practices in the library, benefiting all users. As an open and freely available project, we would be thrilled to serve as a foundation for future browser-based research.

## Appendix: Validation study methods

For many experimental paradigms, the accuracy of stimulus’ presentation durations and the measurement of response times is paramount. To rigorously assess the performance of lab.js, we therefore captured the generated stimulus durations and simulated response times of different lengths precisely using external hardware.

A simple experiment alternated screens of dark and light stimuli. To test display durations, 100 stimuli of either brightness were shown in succession, set to a timeout of either 50, 100, 250, 500, or 1000 ms. For response time validation, we conducted a second experiment with the same setup that waited for keyboard input on every screen, and we simulated a keypress after each of the aforementioned intervals. We ran this experiment through all presentation and response durations across the most recent versions of all major browsers (Firefox, Chrome, Safari, Internet Explorer Edge) and operating systems (Windows, Ubuntu Linux, Mac OS), on modest hardware (Table 1).

**Table 1:** Hardware used in the timing validation study

	pc	mac
Processor	Intel Core i3	Intel Core i5
Clock frequency	3.5 GHz	2.7 GHz
Memory	8GB	16GB
Graphics adapter		Intel Iris Graphics
Screen resolution	1280 x 720, 60Hz refresh rate	
Operating System	Windows 10	Mac os 10.14
	Ubuntu 18.10	

Specialized video analysis hardware (Nexys Video; Digilent Inc.) decoded the screen content as output by the computer via its HDMI port. We monitored and recorded the brightness of the center pixel, as well as the onset of every frame (vertical sync). To generate responses, a USB development board (Teensy 3.5; PJRC) connected to the computer simulated key presses following an increase in brightness. To verify the software’s frame synchronization, response intervals were timed starting from the frame onset. We captured screen content, frame onsets, and USB data using a logic analyzer (Logic Pro 8; Saleae, Inc.) sampling every 20 ns (50 MHz sampling rate). The complete data and analysis scripts are available via https://lab.js.org/performance/.

## Footnotes

1 Earlier data can, of course, also be accessed, for example to calculate aggregate performance. The data storage mechanism is described in the documentation at https://labjs.readthedocs.io/en/latest/reference/data.html

2 This corresponds to the prepare-run-strategy introduced by Mathôt et al., (2012)

3 The documentation provides an overview of the available styling options at https://labjs.readthedocs.io/en/latest/reference/style.html

4 The idea of generic form processor goes back to Göritz and Birnbaum (2005) and Reips and Neuhaus (2002).

5 A visual questionnaire builder for lab.js is currently under development

6 At the time of writing, lab.js provides direct export to the Netlify hosting service (https://netlify.com).

7 The idea of a generic server-side data collection script was introduced by Birnbaum (2000) and Göritz and Birnbaum (2005).

8 At the time of writing, some browsers add a small amount of artificial jitter—2 ms in the case of Firefox—to all time measurements for security reasons, limiting the potential timing precision of any browser-based data collection project. Having resolved the underlying security issues, the Chrome developers have proceeded to remove this artificial source of noise, and we expect the other browsers to follow suit.

9 At the time of writing, Internet Explorer is slated for replacement by a new browser with the same technical foundation as Chrome

10 Whenever a research question requires exact, absolute interpretation of time measurements, we recommend that the hardware and software setup be subjected to careful timing validation. Having performed such a validation and replicated our results, for example, a researcher might confidently subtract any consistent browser-specific lag from all measured response times.

11 A full code-based Stroop task is available from our repository at https://github.com/felixhenninger/lab.js/tree/master/tasks
